# Faunistic Study of the Rodents of North Khorasan Province, North East of Iran, 2011–2013

**Published:** 2018-06-13

**Authors:** Kourosh Arzamani, Zeinolabedin Mohammadi, Mohammad Reza Shirzadi, Seyed Mohammad Alavinia, Behruz Jafari, Jamshid Darvish

**Affiliations:** 1 Vector-Borne Diseases Research Center, North Khorasan University of Medical Sciences, Bojnurd, Iran; 2 Department of Biology, Faculty of Sciences, Ferdowsi University of Mashhad, Mashhad, Iran; 3 Zoonoses Control Department, Ministry of Health, Tehran, Iran; 4 Environmental Office of North Khorasan Province, Bojnurd, Iran; 5 Rodentology Research Department, Applied Animal Institute, Ferdowsi University of Mashhad, Mashhad, Iran

**Keywords:** Fauna, Rodents, Biodiversity, Iran

## Abstract

**Background:**

Rodents are one of the most important hosts for some zoonotic diseases and also act as a reservoir of some ectoparasites and endoparasites. They cause damage to the farms and inflict public health. The aim of this study was to investigate the faunal composition of rodent in North Khorasan Province, Northeast of Iran.

**Methods:**

The sampling was carried out between 2011 to 2013. The specimens were collected using different methods including rodent death and live traps, digging of their burrow, and hand net from 75 different sample sites.

**Results:**

The total number of 396 specimens belonging to 22 species and six families were identified. The results illustrated the high numbers and densities of *Meriones persicus* (17.68%), *Meriones libycus* (15.15%), *Nesokia indica* (7.32%) and *Rhombomys opimus* (6.82%), as the most important reservoirs for different zoonotic diseases. Moreover, significant number of other rodent species including *Mus musculus* (15.66%), *Apodemus witherbyi* (13.89%), *A. hyrcanicus* (0.25%), *Rattus norvegicus* (1.01%), *Meriones crassus* (0.25%), *Gerbillus nanus* (0.51%), *Microtus paradoxus* (2.27%), *M. transcaspicus* (0.76%), *Ellobius fuscocapillus* (0.25%), *Cricetulus migratorius* (4.29%), *Calomyscus elburzensis* (4.29%), *C. mystax* (1.26%), *Spermophilus fulvus* (0.25%), *Dryomys nitedula* (3.54%), *Allactaga elater* (3.54%), *Jaculus blanfordi* (0.25%), *Meriones zarudnyi* (*0.25%*), *M. meridianus* (*0.51%*), and *Hystrix indica* as hosts for parasites and zoonotic diseases were identified.

**Conclusion:**

The high biodiversity including at least 22 species and six families of rodents were found in North Khorasan Province, some of them were medically important species.

## Introduction

Rodents are the most widely distributed and the largest group of small mammals’ worldwide (1) which cause economic loss and inflict public health. Rodents play a significant role as reservoirs or vectors of sixty different diseases including leishmaniasis, leptospirosis, plague, Hantavirus Pulmonary Syndrome, salmonellosis, etc. with direct or indirect role in spread of these diseases (2).

The fauna of the rodent is medically important in Iran and in North Khorasan Province (3–8). These are identified and reported 15 species of rodent from this Province, *Spermophilus fulvus*, *Microtus transcaspicus*, *M. paradoxus*, *Ellobius fuscocapillus*, *Cricetulus migratorius*, *Calomyscus* sp., *Mus musculus*, *Apodemus witherbyi*, *Nesokia indica*, *Gerbillus nanus*, *Meriones libycus*, *M. meridianus*, *M. persicus*, *Rhombomys opimus* and *Dryomys nitedula* (7).

North Khorasan Province was formerly known as eastern range of human plague epidemics in Iran (5) and also has been the objective for study of rodent-borne diseases, especially, as a purpose of investigation on Leishmaniasis and leptospirosis (6, 9). The province is bordered by Golestan Province in the west which is one of the most important focus of zoonotic cutaneous leishmaniasis in Iran (3).

The province is located near the border of Turkmenistan and Afghanistan countries. Neighbour regions in Turkmenistan are mainly deserts and semi-desert areas and zoonotic cutaneous and visceral leishmaniasis are endemic in the country (10). Both of these low-income countries receive low health care and public services; therefore the province can be a trajectory for transportation of infectious disease from both countries via communications and transportation of nomads, refugees, and passengers. North Khorasan Province is one of the most important areas for agriculture and animal husbandry in Iran and the study area is potentially provide a suitable habitat for distribution of pests and favoring habitats for endemic and penetrating species such as Jirds and gerbils. This increases the risk of spreading vector-borne diseases and zoonosis, therefore, this province needs more consideration by Iranian health care service to control and preventing the spread of rodent-borne diseases.

We aimed to investigate the faunal composition of rodents in North Khorasan Province, Northeast of Iran.

## Materials and Methods

### Study area

**Fig. 1.**
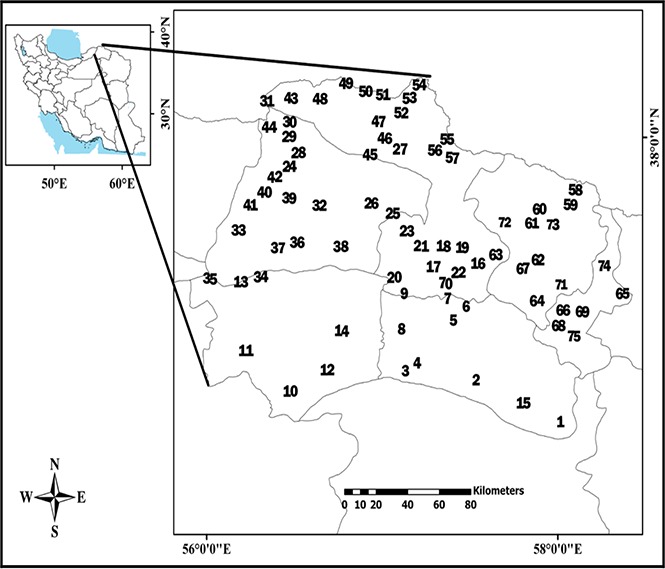
Map of the study area. The samples were taken from 75 regions marked by a number from North Khorasan Province, Iran, 2011–2013

This cross-sectional study was conducted from 36°37′–38°17′ N latitude and 55°53′–58°20′ E longitude in North Khorasan Province, Northeast of Iran with the total area of approximately 28434km^2^. The sampling was carried out between 2011 to 2013 and includes 75 localities of eight counties from North Khorasan Province (Table 1, Fig. 1).

**Table 1.** Geographical coordinates and sampling locality of the rodents of North Khorasan Province, Iran, 2011–2013

**Town**	**Sampling Locality**	**Number of Locality on the map**	**Latitude**	**Longitude**
**Esfarayen**	Safi Abad	1	36°39′ 01.93	58°00′ 19.05
	Gerati	2	36°51′ 53.88	57°31′ 40.58
	Kal Shor	3	36°53′ 51.22	57°07′ 52.74
	Gorpan	4	36°56′ 46.82	57°12′ 16.15
	Esfraien	5	37°08′ 43.87	57°24′ 13.20
	Roein	6	37°12′ 38.02	57°28′ 21.97
	Emam Vardi	7	37°15′ 04.35	57°22′ 30.77
	Jafar Abad	8	37°06′ 17.54	57°06′ 24.94

**Bojnurd**	Salook	9	37°16′ 32.15	57°08′ 07.37
	Esfidan, Chenaran	16	37°24′ 49.70	57°33′ 00.02
	Mehnan, Metranloo	17	37°23′ 51.17	57°17′ 23.46
	Baghchegh	18	37°29′ 27.74	57°21′ 02.96
	Charkharvar, Peighoo	19	37°28′ 58.48	57°27′ 38.07
	Salook 2	20	37°20′ 40.92	57°03′ 58.60
	Bidak	21	37°29′ 27.74	57°13′ 29.32
	Darsofian, Abchoor	22	37°22′ 23.36	57°26′ 10.27
	Tatar	23	37°33′ 51.16	57°08′ 36.64
	Turkmenistan border	55	37°59′ 42.33	57°22′ 01.50
	Jodar	56	37°56′ 17.46	57°18′ 07.36
	Gifan	57	37°54′ 35.02	57°23′ 58.57
	Sisab	63	37°27′ 01.40	57°39′ 05.86
	Asadli	70	37°19′ 42.39	57°21′ 46.86

**Jajarm**	Miandasht	10	36°48′ 43.91	56°29′ 05.97
	Kal Shor	12	36°54′ 20.48	56°41′ 17.66
	Gamiteh	14	37°05′ 04.37	56°46′ 24.97
	Esmaeilabad	15	36°44′ 49.76	57°48′ 07.31

**Garmeh**	Kal Yazd, Daraq	11	36°59′ 44.42	56°13′ 29.42
	Sharlegh	13	37°18′ 58.49	56°12′ 01.61
	Robat Gharahbil	34	37°20′ 40.92	56°18′ 51.36
	Golestan National Park	35	37°20′ 11.66	56°01′ 17.73

**Mane & Samalqan**	Tazeyab	24	37°51′ 54.08	56°28′ 22.07
	Kikanloo	25	37°38′ 29.19	57°03′ 58.60
	Pish Ghaleh	26	37°41′ 54.06	56°56′ 24.95
	Kohghale	28	37°56′ 32.08	56°31′ 46.96
	Yomogh	29	38°00′ 40.86	56°27′ 52.80
	Kohne Jolge	30	38°04′ 49.63	56°28′ 36.70
	Ashkhaneh	32	37°40′ 40.89	56°38′ 51.32
	Behkade	33	37°33′ 51.15	56°11′ 17.71
	Zard	36	37°30′ 11.64	56°31′ 03.04
	Kastan	37	37°29′ 27.74	56°24′ 27.93
	Ghale-khan	38	37°29′ 57.01	56°45′ 55.70
	Kohe Garmab	39	37°43′ 21.87	56°28′ 22.07
	Garmab	40	37°45′ 18.94	56°19′ 49.89
	River Garmab	41	37°41′ 24.30	56°15′ 26.48
	Amand	42	37°49′ 13.08	56°23′ 29.40
	Kheshtli	44	38°03′ 21.88	56°21′ 46.96

**Raz and Jargalan**	Raz	27	37°56′ 46.72	57°06′ 10.30
	Ayri Qayeh	31	38°10′ 55.48	56°20′ 33.79
	Hesarcheh	43	38°10′ 55.47	56°29′ 20.60
	Tangeh Turkeman	45	37°55′ 48.18	56°56′ 10.32
	Goinik	46	37°59′ 56.96	57°01′ 17.63
	Baghlegh, Ashrafdare	47	38°05′ 18.90	56°59′ 05.93
	Yekeh Suod	48	38°10′ 55.48	56°38′ 51.32
	Bahar, Ghoridare	49	38°15′ 18.88	56°47′ 52.77
	Kalatekariz	50	38°13′ 07.18	56°54′ 27.88
	Sangsar	51	38°12′ 08.65	57°00′ 33.73
	Gholaman	52	38°07′ 45.24	57°06′ 39.57
	Sokhso Enghelab	53	38°11′ 54.01	57°09′ 35.17
	Takaleghoz	54	38°15′ 33.52	57°12′ 45.42

**Shirvan**	Gholool Sarani	58	37°45′ 33.57	58°05′ 55.57
	Ghaltmanlo	59	37°41′ 24.80	58°04′ 27.77
	Palkanlou	60	37°39′ 56.99	57°53′ 29.25
	Gholanloo	61	37°35′ 48.22	57°51′ 17.55
	Shirvan	62	37°25′ 33.60	57°52′ 58.98
	Gelian	64	37°13′ 51.18	57°52′ 59.98
	Hossein Abad	67	37°22′ 52.63	57°47′ 52.67
	Devin	71	37°19′ 13.12	58°01′ 17.53
	Topkanloo	72	37°36′ 32.12	57°42′ 01.46

**Farouj**	Titkanloo	65	37°16′ 02.88	58°22′ 30.66
	Mayvan	66	37°11′ 10.21	58°02′ 01.43
	Khosravieh	68	37°07′ 01.44	58°00′ 18.99
	Cheri	69	37°10′ 40.94	58°08′ 36.54
	Barzoo	73	37°35′ 33.58	57°58′ 36.59
	Koran Kordieh	74	37°23′ 36.52	58°15′ 40.92
	Sogtali, Zinkanloo	75	37°04′ 05.84	58°05′ 26.30

### Rodent collection and Methods

The specimens were collected using different methods including live trap, rodent death trap, digging into their burrow, and hand net during all seasons. Geographical coordinates were recorded using GPS. Besides, 11 specimens of rodents deposited in the Zoology Museum of Ferdowsi University of Mashhad (ZMFUM), Mashhad, Iran from this region were considered (Table 2).

**Table 2.** Abundance, percent and different habitats of the rodents of North Khorasan Province, Iran, 2011–2013

**Family**	**Species**	**Locality number**	**N**	**%**	**Collection habitats**										

					**A**	**B**	**C**	**D**	**E**	**F**	**G**	**H**	**I**	**J**	**K**
**Muridae**	*M. musculus*	4, 5, 7, 13, 15, 16, 18, 19, 21, 25, 34, 29, 46, 47, 49, 50, 51, 54, 57, 60, 69, 70	62	15.66		×		×					×		
	*A. witherbyi*	5, 6, 7, 11, 14, 15, 16, 17, 19, 22, 23, 25, 28, 31, 35, 36, 39, 48, 49, 51, 3, 54, 58, 59, 60, 61, 63, 68, 70	55	13.89	×		×	×	×		×	×	×	×	×
	*A. hyrcanicus*	59	1	0.25								×			
	*N. indica*	9, 20, 28, 29, 30, 38, 60, 51, 52	29	7.32		×							×	×	
	*R. norvegicus*	38	4	1.01		×									
	*R. opimus*	3, 4, 34	27	6.82					×						
	*M. libycus*	1, 2, 3, 4, 11, 14, 22, 26, 32, 33, 34, 39, 40, 41, 42, 46, 55, 60, 63, 65, 66, 71	60	15.15	×			×	×		×		×	×	
	*M. persicus*	2, 3, 14, 16, 19, 20, 22, 23, 24, 27, 28, 29, 30, 35, 36, 37, 38, 43, 45, 48, 47, 56, 57, 58, 59, 62	70	17.68	×		×	×	×		×		×	×	×
	*M. crassus*	3	1	0.25					×						
	*M. zarudnyi^1^*	72	1	0.25					×						
	*M. meridianus^2^*	64	2	0.51					×	×					
	*G. nanus^3^*	11	2	0.51						×					

**Cricetidae**	*M. paradoxus^4^*	9, 20, 61, 67, 71	9	2.27	×								×		
	*M. transcaspicus^5^*	67	3	0.76	×								×		
	*E. fuscocapillus*	51	1	0.25									×		
	*C. migratorius*	16, 21, 28, 29, 60, 69	17	4.29									×		

**Calomyscidae**	*C. elburzensis*	16, 20, 36, 37, 42, 53, 58, 61, 64	17	4.29											×
	*C. mystax*	50, 53, 57	5	1.26											×

**Sciuridae**	*S. fulvus*	4	1	0.25					×						

**Gliridae**	*D. nitedula*	16, 28, 35, 44, 61	14	3.54				×					×		

**Dipodidae**	*A. elater*	11, 12	14	3.54						×					
	*J. blanfordi*	10	1	0.25						×					

1. One specimen of *M. zarudnyi* belonged to museum samples.

2. Two specimens of *M. meridianus* belonged to museum samples.

3. One specimen of *G. nanus* belonged to museum samples.

4. Five specimens of *M. paradaxus* belonged to museum samples.

5. Two specimens of *M. transcaspicus* belonged to museum samples.

(LN: Locality Number, A: Pasturage area, B: Urban area, C: Deciduous forest, D: Scrub woodlands, E: Semi desert and Salt land, F: Sandy dunes, G: Steppe scrub lands, H: Juniper’s forest, I: Fields and gardens, J: Adjacent stream and river, K: Mountain area)

Specimen collection was performed in accordance with the procedures approved by the Ethical Committee of North Khorasan University of Medical Sciences.

The materials were identified using available identification keys (11–13). Taxonomic names and distribution of rodents followed Musser and Carleton (14). ArcGIS ver.9.3 software was applied for preparing of the map of sampling localities.

## Results

Overall, 385 collected specimens were investigated. The specimens belong to 22 species, 16 genera, and six families. Of these, *M. percicus*, *M. libycus*, *A. witherbyi* and *M. musculus*, occur in the most localities studied and identified as widely distributed species. Abundance and different habitats of the rodents collected in the study area are shown in the Table. 2. All specimens of *Apodemus* captured from different localities of the province were identified as *A. witherbyi* based on morphology and morphometric approach. Only one *A. hyrcanicus* specimen from Shirvan has been identified. Specimens of *R. opimus* were diagnosed as *R. opimus sargadensis* based on the morphology and morphometric studies. Tracks, spines and some live specimens of *Hystrix indica* were observed in both arid regions and deciduous forests, but we avoided trapping of this species.

## Discussion

This is the first study that reports *Rattus norvegicus*, *M. crassus*, *Allactaga elater*, *Jaculus blanfordi* and *H. indica* in North Khorasan.

Extensive distribution of fields and pasturage in the study region provides suitable habitat for the existence of different kinds of rodents. In the present study, *J. blanfordi* captured from sand dunes and *M. crassus* trapped in semi-desert and salt lands were new records from the region. This species was previously reported from southern parts of Iran, and a new additional record was added from North Khorasan to its occurrence range. *Meriones crassus* is distributed in south-western Palaearctic (14) and in this study, we reported it from the North-Eastern part of its distributional range.

*Rattus norvegicus* had been reported only from Mashhad in Northeast of Iran (7) and this study is the first record of the species from North Khorasan Province. *Allactaga elater* reported previously from South Khorasan and Khorasan Razavi (7) and we reported this species from North Khorasan for the first time. *Meriones persicus*, *M. libycus*, *M. musculus*, *N. indica*, and *R. opimus* were the most abundant rodent species, especially in rural areas. These species are common both in Iran and Turkmenistan. Central parts of Iran demonstrated actually different fauna from Turkmenistan region, however, northeast of Iran (Khorasan) was affected by both cradles in species composition and also correlated to endemism (15).

In total, genus *Meriones* is known as a natural reservoir of *Yersinia pestis* in endemic foci of plague in Iran and genus *Microtus*, *Mesocricetus*, *Allactaga*, *Cricetulus* play a minor role (16). *Mus musculus* (reservoir of *Hymenolepis* and *Syphacia*) and *N. indica* (a secondary host for *Leishmania turanica*) are rodents with zoonotic importance (17, 18) and were captured in high numbers through the northern and central parts of the province in farms and rural areas near the streams. *Apodemus witherbyi* were also one of the most abundant rodents in most of the habitats except very dry sand dunes and urban areas. The wood mice of the genus *Apodemus* collected from most of North Khorasan with relatively high level of humidity were considered to be the vector for *Babesia*, *Hepatozoon*, *Trypanosoma*, and *Grahamella* (19). *Dryomys nitedula* are distributed in gardens and forests around villages in the North Khorasan Province. *Dryomys nitedula*, *Microtus*, *Mus*, and *Calomyscus* have recently demonstrated positive results for *Francisella tularensis* and can play a role to transmit tularemia in Iran (20).

In this study, some of the most important reservoirs of leishmaniasis, leptospirosis, tularemia, plague and other well-known reservoirs of zoonotic diseases were collected. The most records of cutaneous leishmaniasis in North Khorasan Province in the recent years were from rural areas of Garmeh, Jajarm, Bojnurd, and Esfarayen (21), which is consistent with our results of collecting the highest number of *M. libycus*, *M. persicus* and *R. opimus* as the most well-known hosts of the cutaneous leishmaniasis. Previously, *R. opimus* was reported as a host of *L. major* in Esfarayen (9) county and Golestan Province (adjacent province to the North Khorasan) (22).

North Khorasan Province is one of the most important foci of visceral leishmaniasis in Iran (23) and the disease agent was isolated from *M. persicus* and *C. migratorius* (24) while, *M. persicus* was one of the most abundant rodent species and significant numbers of *C. migratorius* were captured in the province.

*Rattus norvegicus* collected from North Khorasan Province, was previously reported as one of the reservoirs of *Giardia muris* (protozoan) and some other parasites in Ahvaz, Southwest of Iran (25) and leptospirosis (6, 26). Regarding to increasing population of Norwegian rats in the cities, their population should be controlled to prevent potential risk to public health.

## Conclusion

Our results demonstrate the high biodiversity of rodents in North Khorasan Province of Iran. *Meriones percicus*, *M. libycus*, *A. witherbyi* and *M. musculus*, are present in most localities. Their massive presence in this region is important medically and agriculturally. The present study is a contribution to the ecological study of rodents in this region and an initiation to medically aspects of rodents as the reservoir of some important zoonotic diseases in the Northeast of Iran.

## References

[1] Etemad E (1967) Mammals of Iran. Vol. 1 (Rodents and key to their identification). National Society of Natural Sources and Human Environment Protection Publications, Tehran, p. 288.

[2] Meerburg BG Singleton GR Kijlstra A (2009) Rodent-borne diseases and their risks for public health. Crit Rev Microbiol. 35(3): 221–270. 19548807 10.1080/10408410902989837

[3] Akhoundi M Mohebali M Asadi M Mahmodi MR Amraei K Mirzaei A (2013) Molecular characterization of *Leishmania* spp. in reservoir hosts in endemic foci of zoonotic cutaneous leishmaniasis in Iran. Folia Parasitol. 60 (3): 218–224. 23951928 10.14411/fp.2013.024

[4] Arzamani K Salehi M Mobedi I Adinezade A Hasanpour H Alavinia M Darvish J Shirzadi MR Mohammadi Z (2017) Intestinal Helminths in Different Species of Rodents in North Khorasan Province, Northeast of Iran. Iran J Parasitol. 12(2): 267–273. 28761488 PMC5527038

[5] Azizi MH Azizi F (2010) A history of the human plague in Iran. Arch Iran Med. 13(6): 563–569. 21039018

[6] Darvish J Arzamani K Abdolahpoor G Shirzadi M Mohammadi Z Alavinia M (2016) Rodent leptospirosis in North Khorasan Province, Northeast of Iran. Int J Infect Dis. 45: 465.

[7] Darvish J Siahsarvie R Mirshamsi O Kayvanfar N Hashemi N Sadeghi Shakib F (2007) Diversity of the rodents of northeastern Iran. Iran J Anim Biosyst. 2(1): 57–76.

[8] Kassiri H Javadian E Abdigoudarzi M (2011) Natural Leishmania Infection in *Meriones hurrianae* and *Tatera indica* (Rodentia: Cricetidae: Gerbillinae) in Sistan-Baluchistan Province, South-Eastern of Iran. Adv Stud Biol. 3(6): 247–256.

[9] Javadian E Nadim A Tahvildare-Bidruni GH Assefi V (1976) Epidemiology of cutaneous leishmaniasis in Iran: B. Khorassan Part V: Report on a focus of zoonotic cutaneous leishmaniasis in Esferayen. Bull Soc Pathol Exot Filiales. 69(2): 140–143. 1037090

[10] PonirovskiÄ EN Darchenkova NN (2008) Landscape-epidemiological zoning of Turkmenistan by leishmaniasis. Med Parazitol (Mosk). (1): 27–30. 18365471

[11] Corbet GB (1978) The mammals of the Palaearctic region: a taxonomic review. Br Mus p. 314.

[12] Kryštufek B Vohralík V (2005) Mammals of Turkey and Cyprus. Rodentia I: Sciuridae, Dipodidae, Gliridae, Arvicolinae. Vol. 2 Knjiñnica Annales Majora, Koper Univerza na Primorskem p. 292.

[13] Kryštufek B Vohralík V Janžekovič F (2009) Mammals of Turkey and Cyprus: Rodentia II: Cricetinae, Muridae, Spalacidae, Calomyscidae, Capromydae, Hystricidae, Castoridae. Vol. 3 Univerza na Primorskem, Znanstveno-raziskovalno središče, Založba Annales p. 372.

[14] Carleton MD Musser GG (2005) Order Rodentia. In: Wilson DE Reeder DM (Eds): Mammal species of the World, a taxonomic and geographic reference. The John Hopkins University Press, Baltinore, Maryland, pp. 745–1599.

[15] Misonne X (1959) Analyse zoogéographique des mammifères de l’Iran. Institut Royal des Sciences Naturelles de Belgique pp. 1–157.

[16] Karimi Y Rodrigues de Almeida C Petter F (1976) Note sur les rongeurs du nord-est du Brésil. Mammalia. 40 (2): 257–266.

[17] Hajjaran H Mohebali M Alimoradi S Abaei M Edrissian GH (2009) Isolation and characterization of pathogenic *Leishmania turanica* from *Nesokia indica* (Rodentia, Muridae) by PCR-RFLP and ITS1 sequencing in Iran. Trans R Soc Trop Med Hyg. 103(11): 1177–1179. 18829057 10.1016/j.trstmh.2008.08.016

[18] Yousefi A Eslami A Mobedi I Rahbari S Ronaghi H (2014) Helminth infections of house mouse (*Mus musculus*) and wood mouse (*Apodemus sylvaticus*) from the suburban areas of Hamadan City, western Iran. Iran J Parasitol. 9(4): 511–518. 25759732 PMC4345090

[19] Turner C (1986) Seasonal and age distributions of Babesia, Hepatozoon, *Trypanosoma* and *Grahamella* species in *Clethrionomys glareolus* and *Apodemus sylvaticus* populations. Parasitology. 93(Pt 2): 279–289. 3537923 10.1017/s0031182000051453

[20] Mostafavi E Shahraki AH Japoni-Nejad A Esmaeili S Darvish J Sedaghat MM Mohammadi A Mohammadi Z Mahmoudi A Pourhossein B Ghasemi A Gyuranecz M Carniel E (2017) A Field Study of Plague and Tularemia in Rodents, Western Iran. Vector Borne Zoonotic Dis. 17(4): 247–253. 28165869 10.1089/vbz.2016.2053

[21] Rajabzadeh R Arzamani K Shoraka H Riyhani H Hosseini SH (2015) Epidemiological survey and geographical distribution of cutaneous Leishmaniasis in North Khorasan Province, 2006–2013. Int J Epidemiol Res. 2 (4): 197–203.

[22] Mirzaei A Rouhani S Taherkhani H Farahmand M Kazemi B Hedayati M Baghaei A Davari B Parvizi P (2011) Isolation and detection of *Leishmania* species among naturally infected *Rhombomis opimus*, a reservoir host of zoonotic cutaneous leishmaniasis in Turkemen Sahara, North East of Iran. Exp Parasitol. 129(4): 375–380. 21945269 10.1016/j.exppara.2011.08.020

[23] Arzamani K (2012) Visceral leishmaniasis in North Khorasan Province, north east of Iran. Int J Infect Dis. 16: e340–e341.

[24] Mohebali M (2013) Visceral leishmaniasis in Iran: review of the epidemiological and clinical features. Iran J Parasitol. 8(3): 348. 24454426 PMC3887234

[25] Kia E Homayouni M Farahnak A Mohebali M Shojai S (2001) Study of endoparasites of rodents and their zoonotic importance in Ahvaz, south west iran. Iran J Public Health. 30(1–2): 49–52.

[26] Esfandiari B Pourshafie MR Gouya MM Khaki P Mostafavi E Darvish J Bidhendi SM Hanifi H Nahrevanian H (2015) An epidemiological comparative study on diagnosis of rodent leptospirosis in Mazandaran Province, northern Iran. Epidemiol Health. 37: e2015012. 25773440 10.4178/epih/e2015012 PMC4430762

